# Patterns of human-wildlife conflict and compensation practices around Daxueshan Nature Reserve, China

**DOI:** 10.24272/j.issn.2095-8137.2018.056

**Published:** 2018-05-31

**Authors:** Cheng Huang, Xue-You Li, Liu-Jun Shi, Xue-Long Jiang

**Affiliations:** 1 Kunming Institute of Zoology, Chinese Academy of Sciences, Kunming Yunnan 650223, China; 2 Kunming College of Life Sciences, University of Chinese Academy of Sciences, Kunming Yunnan 650223, China; 3 Daxueshan Nature Reserve, Yongde Yunnan 677600, China

**Keywords:** Human-wildlife conflict, Asiatic black bear, Spatial heterogeneity, Insurance scheme, Daxueshan Nature Reserve

## Abstract

Understanding the spatial patterns of human-wildlife conflict is essential to inform management decisions to encourage coexistence, but it is constrained by the lack of spatially-explicit data. We collected spatially-implicit data of human-wildlife conflicts from 2009–2015 around Daxueshan Nature Reserve, Yunnan, China, and investigated the patterns and drivers of these conflicts. A questionnaire was also designed to capture local resident attitudes toward insurance-based compensation for the losses caused by targeted wildlife. We found that the Asiatic black bear (*Ursus thibetanus*) was the most conflict-prone animal around the reserve, followed by the rhesus macaque (*Macaca mulatta*) and Southeast Asian sambar (*Cervus equinus*). Conflicts were unevenly distributed among seasons, villages, and communities, with several grids identified as conflict hotspots. Poisson models revealed that human-bear conflicts were negatively related to distance to the reserve and proportion of forest, but positively correlated to the proportion of cropland. Binomial models showed that communities affected by crop depredation were positively correlated with the proportion of cropland and negatively correlated with distance to the reserve, whereas communities affected by livestock depredation were negatively correlated with the proportion of cropland. The insurance-based scheme has compensated over 90% of losses, to the satisfaction of 90.6% of respondents. Our results suggest that human-bear conflict could be potentially reduced by eliminating food crops near the reserve boundary and livestock grazing at conflict hotspots. In addition, the insurance-based scheme could be replicated at a broader scale with improvement in loss assessment.

## INTRODUCTION

Human-wildlife conflict, which is defined as any interactions leading to negative impacts on the humans or wildlife involved (Pettigrew et al., 2012), is a worldwide conservation issue. Large- and medium-sized mammals are the primary animals of concern and include species such as lions (*Panthera leo*), snow leopards (*Panthera uncia*), and Asian elephants (*Elephas maximus*) (Bagchi & Mishra, 2006; Chen et al., 2013; Distefano, 2005; Maclennan et al., 2009), which are highly valued by international tourists and researchers. However, local residents often experience variously negative impact due to the presence of wildlife, including crop raiding, livestock depredation, and human casualties (Dickman et al., 2011). In turn, local resident hostility can increase, thereby threatening wildlife conservation (Dickman, 2010).

Prevention before conflict and mitigation after conflict are two general strategies used to tackle human-wildlife conflict (Marchal & Hill, 2009; Mishra et al., 2003; Pettigrew et al., 2012). Prevention is widely recognized as the better strategy (Goodrich, 2010; Treves & Karanth, 2003), and includes guarding and fencing of livestock, zoning of land, and increasing prey abundance for carnivores (Guo et al., 2012; Mishra et al., 2003). Determining the areas in which to implement such measures is the first concern, and thus understanding the spatial heterogeneity of conflict is the cornerstone to achieve cost-efficient prevention (Dickman et al., 2011). However, studies on spatial heterogeneity are limited by the lack of long-term spatially-explicit conflict data, which require extensive labor to collect and standard approaches to assess (Pettigrew et al., 2012). Therefore, conflict mitigation measures are still widely used (Li et al., 2013; Mishra et al., 2003), among which monetary compensation is the most common. As a result, spatially-implicit conflict data are well documented by compensation schemes, which could allow promising insight into the spatial patterns of human-wildlife conflict (Chen et al., 2016b).

In China, compensation schemes are funded by several levels of government, whereby monetary compensation is offered for the losses caused by targeted animals listed as nationally protected species under the National Wildlife Law. Within Yunnan, the first scheme was funded by the provincial government from 1999–2008 and the second scheme was funded by the state government from 2009–2013. To date, the insurance-based scheme is the most expansive, and was initiated in 2007 to mitigate human-elephant conflict at Xishuangbanna, Yunnan, China. In this scheme, adjustors from the insurance company assessed the losses and recorded the conflict data, including the household affected, date of conflict, wildlife involved, type of damage, conflict locality, loss assessment, and compensation amount. Previous studies have reported that a high proportion of local residents were dissatisfied with this scheme after suffering significant losses caused by Asian elephant in south Yunnan and large-sized carnivore in the Qinghai-Tibetan Plateau (Chen et al., 2013, Chen et al., 2016a). However, local resident attitudes toward this scheme with different conflict-prone animals as well as conflict severity remain poorly evaluated.

Here, we aimed to (1) identify the hotspots of human-wildlife conflict using spatially-implicit compensation data, (2) investigate factors potentially affecting the patterns of human-wildlife conflict, and (3) evaluate the attitudes of local residents toward insurance-based compensation schemes.

## MATERIALS AND METHODS

### Study area

This study was conducted around the Daxueshan Nature Reserve (DNR), Yunnan, China (175.41 km${}^{2}$, E99${}^{\circ }$32${}^{\prime }$–99${}^{\circ }$43${}^{\prime }$ and N24${}^{\circ }$00${}^{\prime }$–24${}^{\circ }$12${}^{\prime }$). The elevation within the reserve ranges from 960 m to 3 504 m a.s.l., with diverse vegetation ranging from tropical seasonal rainforest to temperate alpine forest. Large- and medium-sized mammals are found within DNR, including the western black-crested gibbon (*Nomascus concolor*), northern pig-tailed macaque (*Macaca leonina*), rhesus macaque (*Macaca mulatta*), Southeast Asian sambar (*Cervus equinus*), Indian muntjac (*Muntiacus vaginalis*), wild boar (*Sus scrofa*), and Asiatic black bear (*Ursus thibetanus*) (Guo, 2006). In the area surrounding DNR, the predominant income of residents comes from cash crops (e.g., walnut, tobacco, and sugarcane) and livestock (e.g., goat and cattle).

### Data collection

We collected human-wildlife conflict compensation data recorded by the DNR and insurance company from January 2009 to September 2015, which consisted of 1 637 compensated conflicts. We assigned conflicts during 2014–2015 into 1-km${}^{2}$ grids by interviewing households in communities with the highest losses. High-resolution landscape images were loaded into Google Earth, then overlaid with the 1-km${}^{2}$ grids labeled with localities. We also designed a questionnaire to collect information on local resident perceptions toward the insurance-based scheme (Supplementary Table S1). We collected socioeconomic information during 2009–2014 from the Digital Village of Yunnan (http://www.ynszxc.gov.cn/) established by the Yunnan Provincial Government.

We visually interpreted 360 points on the high-resolution imagery to develop a land-use map (Wang et al., 2016). We classified land use into five categories: forest, shrubland, cropland, construction site, and water body.

### Land use classification

Two Landsat 8 OLI_TIRS images captured in the dry season in 2015 were used to develop the land-use map. We pre-processed the Landsat images as per Young et al. (2017). In addition to the spectral information provided by image bands, ancillary data were added to improve classification accuracy, including the Normalized Difference Vegetation Index (NDVI), NDVI texture, Digital Elevation Model (DEM), and slope. In total, 240 training points were used to perform supervised classification using a random forest algorithm with the help of the RStoolbox package in R (Leutner et al., 2017; R Development Core Team, 2011). The overall classification accuracy was 0.94 validated by 120 points.

### Statistical analysis

Pre-analysis showed that loss was highly correlated with the number of conflicts (Bear-Livestock: r${}^{2}$=0.89, *P*<0.01; Bear-Crop; r${}^{2}$=0.78, *P*<0.01; Macaque-Crop: r${}^{2}$=0.96, *P*<0.01; Sambar-Crop: r${}^{2}$=1, *P*<0.01); hence, both loss and number of conflicts could be used as indicators of human-wildlife conflict. We excluded eight human-injury events from further analysis as they were stochastic and disproportionally accounted for 23.9% of losses. The predation rate of livestock was defined as the number killed divided by fatstock each year.

We performed *Chi*-square tests of independence to examine whether human-wildlife conflict varied among villages, communities, and seasons. We mapped spatial heterogeneity of the losses from 2009 to 2014 with log transformation at the village scale by spatial Kriging interpolation (Chen et al., 2013). The villages were roughly represented by the location of the village committees. We assigned data into 1-km${}^{2}$ grids from 2014 to 2015 to identify conflict hotspots at the grid scale.

We postulated that five factors potentially affected the number of human-bear conflicts (divided into crop depredation and livestock depredation) at the community level from 2009 to 2014, including distance to DNR, forest, shrubland, cropland, and construction site (as proportions within a 2-km radius of each community). Kendall tests confirmed that correlation of the five factors was lower than 0.7. Due to the large variation in the positive count data, we fitted the data with the “hurdle” model, which combined logistic regression for binomial responses (affected or not affected) and zero-truncated Poisson model for count data (Zeileis et al., 2008). Univariate analysis was carried out for the explanatory covariates, followed by multivariate analysis for significant covariates (excluding shrubland and construction sites, *P*<0.05) (Songhurst & Coulson, 2014). Moran’s I test showed no significant spatial autocorrelation in the baseline model. The resulting models were ranked using Akaike’s information criterion corrected for small sample sizes (AICc) with the MuMIn package in R (Burnham & Anderson, 2002).

## RESULTS

### Description of human-wildlife conflict

The 1 526 conflicts caused total economic losses of USD206 341. Asiatic black bear, rhesus macaque, and Southeast Asian sambar were the major animals involved (Table 1), contributing to 98.7% of the conflicts. The Asiatic black bear caused the greatest damage, accounting for 77.7% of conflicts and 88.2% of losses, followed by the rhesus macaque and Southeast Asian sambar (17.0% of conflicts and 8.6% of losses and 4.7% of conflicts and 1.9% of losses, respectively). For conflicts attributed to the Asiatic black bear, livestock depredation caused disproportional losses compared to the frequency of conflicts (49.6% vs. 18.2%), with an average predation rate of 2.57%.

**Table 1:** Summary of human-wildlife conflicts during 2009–2014 around Daxueshan Nature Reserve

Animal	Frequency	Economic loss (US$)	Number of conflicts (n)						
			Maize	Goat	Hive	Bean	Radish	Buckwheat	Other
***Ursus thibetanus***	1 185	181 999	808	278	91	0	0	3	5
***Macaca mulatta***	260	17 736	162	0	0	87	1	0	10
***Cervus equinus***	71	3 941	2	0	0	0	45	17	7
***Psittacula finschii***	6	586	6	0	0	0	0	0	0
**Raptors**	4	2 079	0	0	0	0	0	0	4
**Sum in total**	1 526	206 341	978	278	91	87	46	20	26

### Spatiotemporal pattern of human-wildlife conflict

**Figure 1:**
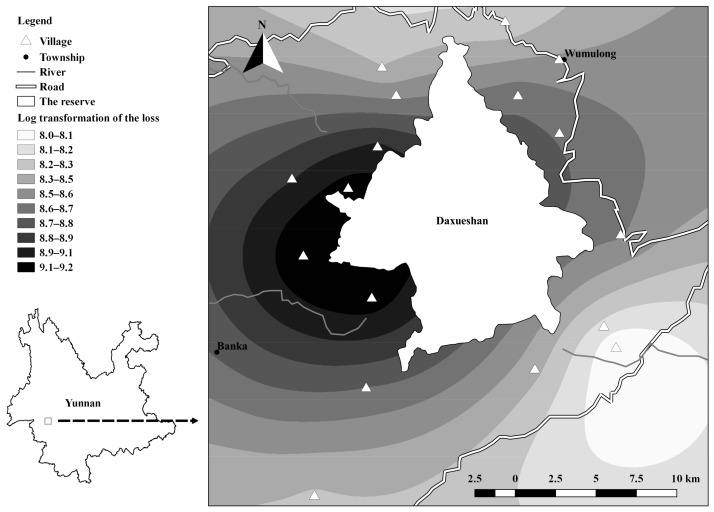
Village-scale risk of conflict around Daxueshan Nature Reserve during 2009–2014

**Figure 2:**
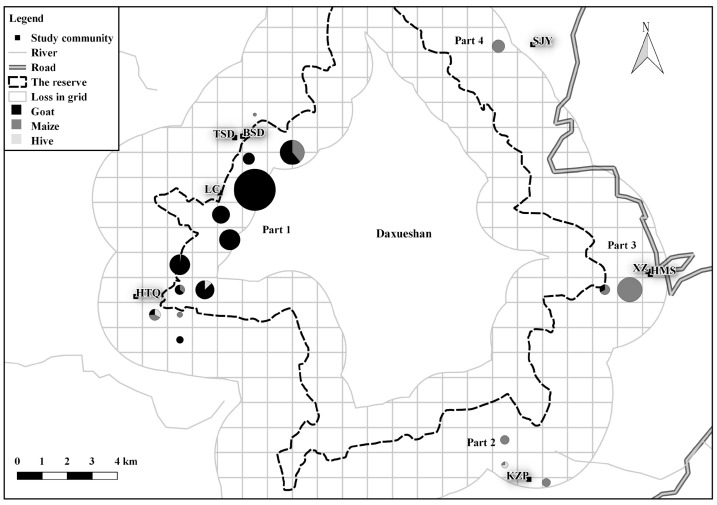
Grid-scale risk of conflict around Daxueshan Nature Reserve during 2014–2015

Conflicts varied among villages and communities (*df*=15, γ${}^{2}$=67.779, *P*<0.01; *df*=76, γ${}^{2}$=231.41, *P*<0.01) and grids (Figure 1, Figure 2). We assigned villages into Parts 1 to 4. Part 1 was comprised of four villages and accounted for 60.5% of losses, with 99.9% caused by the Asiatic black bear, such that the predation rate (9.5%) was much higher than the average. Part 2 was comprised of one village and accounted for 6.5% of losses, with 92.5% attributed to the Asiatic black bear. Part 3 and Part 4 accounted for 9.7% and 10.3% of losses (with 80.6% and 58.6% attributed to the Asiatic black bear), respectively. At the grid scale, 92.6% of conflicts (113 out of 122) were located and assigned to 18 grids (Figure 2). Grids near communities Baishudi (BSD), Taoshudi (TSD), and Luchang (LC) suffered considerably from goat depredation (90.1% of losses). The grids near community Shijiaoyan (SJY) were inhabited by a group of rhesus macaques, which contributed to 99.1% of conflicts. Crop depredation by the Asiatic black bear accounted for the main losses at the grids near communities Kuzhupeng (KZP) (100%) and Xinzhai (XZ) and Huomaoshu (HMS) (95.2%).

Darker gradient represents higher risk.

Pie chart shows proportion of losses. The conflicts of Taoshudi (TSD), Baishudi (BSD), Luchang (LC), Hetaoqing (HTQ), Kuzhupeng (KZP), Xinzhai (XZ), Hongmaoshu (HMS), and Shijiaoyan (SJY) were situated into specific grids.

Of the three predictors (Table 2), the probability of the community suffering cropland depredation was significantly negatively correlated with distance to DNR and positively correlated with the proportion of cropland, whereas the probability of the community suffering livestock depredation was significantly negatively correlated to the proportion of cropland. In the zero-truncated model with positive count data, the number of conflicts, regardless of type of depredation, was significantly negatively correlated with the distance to DNR and proportion of forest, but significantly positively correlated to the proportion of cropland.

**Table 2:** Coefficients of predictors fitted to the probability of the community being affected and number of conflicts

Covariates	Livestock (Binomial)	Livestock (Poisson)	Crop (Binomial)	Crop (Poisson)
**Distance to DNR**	~	–0.77***	–0.95*	–0.31***
**Proportion of cropland**	–0.72*	0. 39***	1.04**	0.14***
**Proportion of forest**	~	–0.42***	~	–0.20***

*: *P*<0.05; **: *P*<0.01; ***: *P*<0.001; ~: *P*≥0.05.

**Figure 3:**
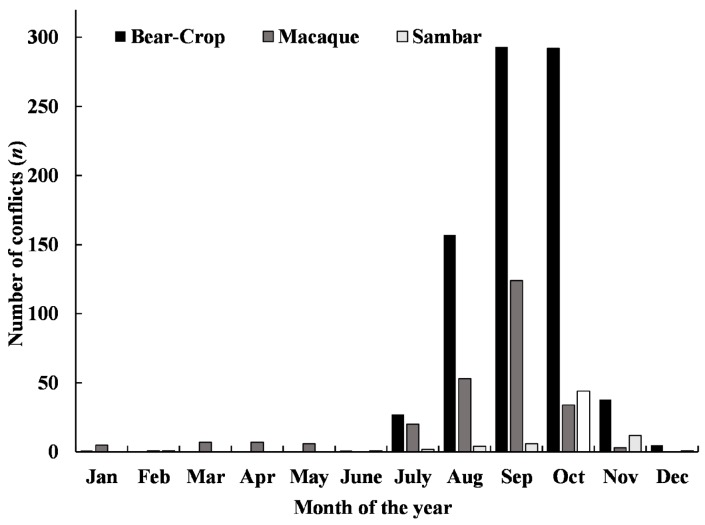
Monthly conflicts of crop depredation caused by Asian black bear, rhesus macaque, and Southeast Asian sambar around Daxueshan Nature Reserve

**Figure 4:**
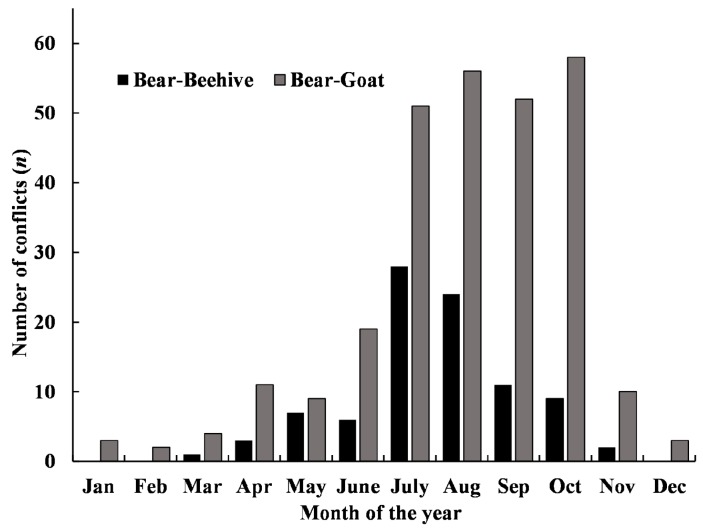
Monthly conflicts involving goats killed and beehives damaged by the Asian black bear around Daxueshan Nature Reserve

The number of conflicts varied among seasons (*df*=3, γ${}^{2}$=19.65, *P*<0.01) (Figure 3, Figure 4). The Asiatic black bear predated goats most frequently from July to October (83.5% of losses) and 99.1% of maize-crop raiding by the black bear occurred from July to November. Rhesus macaques mainly caused damage to maize (62.3% of conflicts) and bean crops (33.5% of conflicts) between July and October. The Southeast Asian sambar caused damage to radish and buckwheat crops, with most of conflicts (78.9%) occurring from October to November.

### Compensation practices and local resident perceptions

We tracked compensation practices back to 2001. The overall compensation ratio (51.1%±28.4%) from 2001 to 2014 fluctuated among schemes, with 72.8% for provincially-funded schemes, 85.3% for state-funded schemes, and 93.4% for insurance-based schemes. The compensation interval was 145±33 days during 2005–2013. The proportion of losses accounting for annual household income dropped from 14.2% to 4.4% from 2009 to 2014.

We collected 53 questionnaires that comprised 35.1% of households involved in conflicts from 2014 to 2015. Results showed that although herds were guarded by herders and kept in enclosures at night, unexpected Asiatic black bear attacks occurred occasionally, with the carcasses of goats subsequently having no market value for the farmers. In total, 90.6% of respondents were generally satisfied with the insurance-based scheme, with 32.1% slightly unsatisfied with the delay or unfair assessment and low compensation ratio. Furthermore, 60.37% of respondents were willing to pay extra insurance premiums for greater compensation. Despite the losses, no respondents had a negative attitude regarding conservation around DNR.

## DISCUSSION

### Implication to prevent human-wildlife conflict

Resources to reduce human-wildlife are limited, so it might not be necessary to allocate significant effort to less conflict-prone animals or areas. The DNR is inhabited by an assemblage of large- and medium-sized mammals, only several of which are involved in human-wildlife conflicts. Among those animals, the Asiatic black bear was identified as the most damaging and tends to be problematic across much of Asia (Escobar et al., 2015; Lewis et al., 2015; Li et al., 2013). Thus, reduction in human-bear conflicts should be addressed first to reduce livestock depredation.

Human-bear conflicts are distinctive events and affected by several factors around DNR, highlighting how the black bear trade-off between resource extraction and mortality risk (Basille et al., 2009; Goswami et al., 2014; Munshi-South et al., 2008). Distance to DNR and proportion of cropland showed consistent impact on the patterns of livestock and crop depredation, both of which were much more frequent from July to November, suggesting that livestock and crop depredation by the Asiatic black bear were synchronously related. During this period, anthropogenic foods, especially maize, are much more available than natural foods, thus impacting Asiatic black bear foraging (Lewis et al., 2015). The negative correlation of distance to DNR revealed that conflicts were partially caused by black bears from within the reserve (Karanth et al., 2013). This is because suitable habitat for large-sized mammals outside the reserve has been replaced by agriculture and infrastructure, with the current human population around DNR being 20 times higher than the population in the 1950s. The proportion of cropland was a major factor reducing the probability of the community being affected by livestock depredation. This may be because the Asiatic black bear potentially avoided predating goats in areas with higher human disturbance, but if anthropogenic crops were available, they would risk foraging on crops and prey on goats at the same time. Thus, we recommend eliminating food crops near the DNR boundary as well as livestock grazing at conflict hotspots and offering intense guarding to reduce human-bear conflicts.

### Improving compensation practices

Although monetary compensation receives mixed outcomes (Pettigrew et al., 2012), it is still a widespread and straightforward approach to satisfy victims of conflict (Dickman et al., 2011). Pettigrew et al. (2012) stated that speed, transparency, funds, separate responsibilities, involvement of experts or trained locals, and clear guidelines are the key components of successful schemes. For the insurance-based schemes, the speed of compensation and transparency were not complained by the respondents around DNR. In addition, a third-party insurance company was in charge of assessing and compensating losses and the market value of the losses was largely compensated. Consequently, the overall attitude toward the scheme was positive, rather than the 20% of market value compensation for rubber losses in human-elephant conflicts and the remoteness and inaccessibility of the Qinghai-Tibet Plateau (Chen et al., 2013; Chen et al., 2016b). Moreover, the proportion of losses accounting for annual household income decreased to one third from 2009 due to the overall increase in income. Our results suggest that the insurance-based scheme could be replicated at a broader scale with improvement in unfair, untimely, and improper assessment. Nevertheless, the insurance-based scheme excluded many animals involved in human-wildlife conflicts before 2015, such as the wild boar and Indian muntjac, despite the wild boar being identified as the most conflict-prone animal following the Asian elephant (*Elephas maximus*), accounting for 38.4% of losses in Yunnan (Pettigrew et al., 2012). Therefore, it is necessary to enlarge the list of animals for which compensation is offered to the victims of conflict.

Currently, the insurance-based scheme is not risk-based and does not include resident participation, which has been criticized (Chen et al., 2013). It has been proposed that a scheme with local participation would ensure more effective management of human-wildlife conflict as it would allow residents to take greater responsibilities (Chen et al., 2013). Our study showed that a high proportion of respondents (60.4%) were willing to pay extra insurance premiums to receive greater compensation; therefore, schemes with local resident participation would improve future compensation.

## CONCLUSIONS

Understanding the spatiotemporal patterns of human-wildlife conflict is imperative for the cost-efficient implementation of avoidance and mitigation measures. We presented the spatial heterogeneity of conflict at an operational scale with spatially-implicit data from conflict compensation schemes. Records from mitigation schemes after conflict could facilitate the prevention of conflict in the first place. However, studying spatial patterns at a finer scale with spatially-implicit data is still challenging; thus, we suggest that spatial information should be recorded by noting the specific site coordinates and dates of conflicts.

## Supplementary Material

http://www.zoores.ac.cn/fileup/2095-8137/SUPPL/20180803181748.zip
